# 

**Published:** 1903-02-28

**Authors:** 


					the hospital. "The standard of highest purity."?Loncet. Feb. 28th, 1903.
CADBURY'S ^ rj CADBURY'S
COCOA M. M COCOA
ABSOLUTELY PURE. c? ? NO DRUOS OR ALKALIES USED.
No. 857, Yol. XXXIII. GfffiSSUV] Saturday,
Feb. 28th, 1903.
[Subscriptior^Terni?,] pRI0H TWOPENCE,

				

